# Serological epitope profile of anti-Ro52–positive patients with systemic autoimmune rheumatic diseases

**DOI:** 10.1186/s13075-015-0871-3

**Published:** 2015-12-17

**Authors:** Maria Infantino, Francesca Meacci, Valentina Grossi, Maurizio Benucci, Gabriella Morozzi, Elio Tonutti, Marilina Tampoia, Antonina Ott, Wolfgang Meyer, Fabiola Atzeni, Piercarlo Sarzi-Puttini, Mariangela Manfredi, Nicola Bizzaro

**Affiliations:** SOS Laboratorio Immunologia e Allergologia, Ospedale S. Giovanni di Dio, Via Torregalli 3, 50143 Firenze, Italy; SOS Reumatologia, Ospedale S. Giovanni di Dio, Firenze, Italy; Dipartimento di Scienze Mediche Chirurgiche e Neuroscienze, Policlinico Le Scotte, Siena, Italy; Immunopatologia e Allergologia, Azienda Ospedaliero-Universitaria, Udine, Italy; Laboratorio di Patologia Clinica, Policlinico Universitario, Bari, Italy; Euroimmun AG, Luebeck, Germany; UOC Reumatologia, Ospedale L. Sacco Polo Universitario, Milano, Italy; Laboratorio di Patologia Clinica, Ospedale San Antonio, Tolmezzo, Italy

**Keywords:** Anti-Ro52 antibodies, Rheumatic diseases, Anti-Ro52 epitopes

## Abstract

**Background:**

Ro52 is an interferon-inducible protein of the tripartite motif family. Antibodies against Ro52 have been described in patients with different autoimmune diseases, such as systemic lupus erythematosus and Sjögren’s syndrome, that are often associated with anti-Ro60 antibodies. The Ro52 autoantigen is extraordinarily immunogenic, and its autoantibodies are directed against both linear and conformational epitopes. The aim of this study was to evaluate the prevalence of antibodies to the five Ro52 domains, as well as to Ro52 176– to 196–amino acid (aa) and 200–239-aa peptides, in different systemic autoimmune rheumatic diseases (SARDs). We also aimed to verify whether antibodies to a single domain or domain association could increase their diagnostic specificity for any SARD.

**Methods:**

Serum samples were obtained from 100 anti-Ro52 antibody–positive patients with SARDs and from 68 controls (50 healthy donors and 18 patients with other autoimmune or allergic diseases). A special line immunoassay was created containing a full-length Ro52 antigen expressed in insect cells using the baculovirus system, five recombinant Ro52 antigen fragments [Ro52-1, Ro52-2, Ro52-3, Ro52-4 (partly overlapping Ro52-1 and Ro52-2), and Ro52-5 (partly overlapping Ro52-2 and Ro52-3)], and two Ro52 peptides (176–196 aa and 200–239 aa), all expressed in *Escherichia coli*.

**Results:**

In patients with SARDs, fragment prevalence rates were as follows: Ro52-1 = 3 %, Ro52-2 = 97 %, Ro52-3 = 0 %, Ro52-4 = 9 %, Ro52-5 = 28 %, Ro52 175–196-aa peptide = 6 %, and Ro52 200–239-aa peptide = 74 %. All control samples were negative for the full-length Ro52 and for the five fragments tested.

**Conclusions:**

The main epitope of the Ro52 antigen was localized on fragment 2 (aa 125–267), and the majority (97 %) of SARD sera had antibodies that target this fragment. As most of the samples were positive for fragment 2 and only some for fragments 4 or 5, which partially overlap fragment 2, it seems that the target epitope is localized in the middle of fragment 2 or in the area between fragments 4 and 5. No antibody against a single epitope or a combination of epitopes was linked to any of the single SARDs.

## Background

Historically, the SSA/Ro antigen was described as a small cytoplasmic ribonucleoprotein complex consisting of two different proteins, one of 52 kDa (Ro52) and the other of 60 kDa (Ro60). Subsequently, novel pathogenic mechanisms and better biochemical characterization led to consideration of Ro52 no longer as an integral part of the SSA/Ro ribonucleoprotein [1–4] but as a separate antigen, even if in about half of cases antibodies to Ro52 co-occur with anti-Ro60 [5].

Although the presence of anti-Ro52 antibodies has been reported in different systemic autoimmune rheumatic diseases (SARDs) [6], the antibody frequency is higher in idiopathic inflammatory myopathy (IIM) [7, 8], Sjögren’s syndrome (SjS), and autoimmune liver diseases [9]. Indeed, the presence of these autoantibodies in patients with autoimmune hepatitis correlates with a worse clinical course [10]. However, these autoantibodies are not completely specific, as they can be detected in other conditions, such as viral infections (e.g., hepatitis C virus) and neoplastic diseases [11], and sometimes in healthy individuals [12, 13].

The diagnostic usefulness of Ro52 monospecificity is controversial because reports in the literature show a low diagnostic value for all autoimmune diseases except IIM [6]. Therefore, anti-Ro52 antibodies have been classified as myositis-associated autoantibodies, being detectable mainly in polymyositis patients with anti-tRNA synthetase syndrome associated with anti-histidyl tRNA synthetase (anti-Jo1) [14, 15].

In patients with SARDs these antibodies are associated with inflammatory lung disease [16], and in anti-Ro52–positive pregnant women antibodies against the 200– to 239–amino acid (aa) sequence of Ro52 can induce fetal cardiac heart block (CHB) and/or cardiac conduction defects because of their arrhythmogenic action on the fetal heart [16–18]. Despite these associations, the real clinical significance of anti-Ro52 antibodies remains partially controversial, and for this reason often their detection does not have a great impact on connective tissue disease diagnosis [19].

**Fig. 1:**
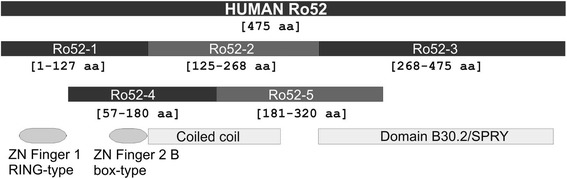
Schematic representation of the Ro52 antigen showing the main immunogenic fragments and structural domains. *aa* amino acid, *ZN* Zinc

The Ro52 antigen, composed of 475 aa, has been identified as an interferon (IFN)-inducible protein of the tripartite motif family of proteins. It has an E3 ligase function that ubiquitinates IFN regulatory factors and proteins. It consists of different domains, including two zinc finger motifs comprising the RING finger, a B-box, a coiled-coil region (CC), and a C-terminal B30.2 domain (PRY/SPRY) [20] (Fig. 1).

According to the literature, the CC domain is the main immunogenic region in patients with systemic lupus erythematosus (SLE), SjS, and IIM. The C-terminal region of Ro52, containing the B30.2 domain, showed higher antibody titers only in patients with SjS [21, 22].

Several laboratory methods can be used to detect antibodies to the full Ro52 molecule (immunoenzymatic assay, line immunoassay, counterimmunoelectrophoresis, Western blot, chemiluminescence, and addressable laser bead immunoassay). Epitope recognition profiles have been analyzed only in anti-Ro52–positive mothers of children with congenital heart block to identify mothers at high risk for having affected children, and it has been suggested that a high ratio between antibody titers to 200–239 aa and 176–196 aa could be used as a marker of heart block risk [16, 17].

This approach stemmed from the observed dominant response to 200–239 aa in all mothers who gave birth to affected children, while antibodies against this peptide were significantly less frequent in mothers who gave birth to healthy children. Instead, sera from the latter mothers reacted mainly with epitopes contained within 176–196 aa of the Ro52 protein.

The aim of our present study was to extend the examination of Ro52 epitope antibody profiles to patients with SARDs and to assess the prevalence of antibodies to the five Ro52 fragments and to Ro52 176–196-aa and Ro52 200–239-aa peptides. Furthermore, as antibodies against the entire Ro52 molecule are not disease-specific, we also aimed to evaluate if the antibodies against any single epitope or a combination of epitopes could have more disease specificity than antibodies against the full-length protein.

## Material and methods

### Patients

Serum samples were obtained from 100 patients with SARDs and from 68 control subjects (50 healthy blood donors and 18 patients with other autoimmune or allergic diseases). The SARD group consisted of 23 patients with SLE, 34 with SjS, 7 with mixed connective tissue disease (MCTD), 27 with IIM, 2 with systemic sclerosis (SSc), and 7 with rheumatoid arthritis. SARD diagnoses were established according to internationally validated disease criteria. Patients with SARDs were selected on the basis of presence of anti-Ro52 antibodies in their sera. Because intermethod variability in measurement of anti-Ro52 antibodies is very high [23–25], Ro52 antibody presence was preliminarily confirmed using four different assays: a BioPlex 2200 antinuclear antibody (ANA) screen (Bio-Rad Laboratories, Hercules, CA, USA) and ANA Profile 3 EUROLine, Autoimmune Liver Disease Profile EUROLine, and Myositis Profile 3 EUROLine line immunoassays (EUROIMMUN, Luebeck, Germany). A serum sample was considered positive when anti-Ro52 antibodies were confirmed by at least three of these methods.

Patient consent was not sought, owing to the retrospective nature of the study and the fact that it was carried out on leftover samples and because all analyses were performed blindly and patients’ records and information remained anonymous.

### Serological assays

A special line immunoassay was created that contained purified full-length Ro52 antigen expressed in insect cells using the baculovirus system and five recombinant Ro52 antigen fragments (Ro52-1–Ro52-5, spanning aa residues 1–127, 125–268, 268–475, 57–180, and 181–320, respectively), according to UniProt accession number P19474, produced in *Escherichia coli*. The immobilized, metal ion affinity chromatography–purified, His-tagged fusion proteins were coated as parallel lines onto nitrocellulose membrane.

Sera were incubated in accordance with the manufacturer’s standard protocol (EUROIMMUN) (30 minutes in serum, 30 minutes in anti-human immunoglobulin G/alkaline phosphatase, and 10 minutes in 5-bromo-4-chloro-3-indolyl-phosphate/nitro blue tetrazolium substrate). Reaction intensities expressed in grayscale units were automatically evaluated using commercially available EUROLineScan software (EUROIMMUN).

### Statistical analysis

Tukey’s method was used to check the data for plausibility and outliers. The statistical evaluation was performed using the Kruskal-Wallis H test and the maximum likelihood estimation. The level of statistical significance was set at α = 0.05. The evaluation was performed using PASW Statistics 17.0 version 10.0.2 software (SPSS, Chicago, IL, USA).

## Results

Ninety-seven (97 %) of one hundred of the samples obtained from the patients with SARDs were positive for antibodies against the Ro52-2 fragment, and all control samples were negative for the full-length Ro52 and for all the fragments except one that was positive for the 175–196-aa and 200–239-aa peptides.

In patients with SARDs, the overall fragment prevalence rates were as follows: Ro52-1 = 3 %, Ro52-2 = 97 %, Ro52-3 = 0 %, Ro52-4 = 9 %, Ro52-5 = 28 %, Ro52 175–196-aa peptide = 6 %, and Ro52 200–239-aa peptide = 74 %. The fragment and peptide prevalence rates in global and single SARD cohorts are described in Table 1.

**Table 1:** Prevalence of antibodies to full-length Ro52 and Ro52 epitopes and peptides in patients with SARDs and controls

	Ro52 epitopes						Ro52 peptides	
	1	2	3	4	5	Full-length Ro52	200–239 aa	175–196 aa
Total SARDs (*n* = 100)	3 (3 %)	97 (97 %)	0	9 (9 %)	28 (28 %)	100 (100 %)	74 (74 %)	6 (6 %)
MCTD (*n* = 7)	0	7 (100 %)	0	0	2 (29 %)	7 (100 %)	5 (71 %)	0
Myositis (*n* = 27)	1 (4 %)	24 (89 %)	0	3 (11 %)	5 (19 %)	27 (100 %)	16 (59 %)	1 (4 %)
RA (*n* = 7)	0	7 (100 %)	0	0	2 (29 %)	7 (100 %)	5 (71 %)	0
SLE (*n* = 23)	1 (4 %)	23 (100 %)	0	1 (4 %)	6 (26 %)	23 (100 %)	19 (83 %)	2 (8 %)
SjS (*n* = 34)	1 (3 %)	34 (100 %)	0	5 (15 %)	13 (38 %)	34 (100 %)	28 (82 %)	3 (9 %)
SSc (*n* = 2)	0	2 (100 %)	0	0	0	2 (100 %)	1 (50 %)	0
Controls (*n* = 68)	0	0	0	0	0	0	1 (1 %)	1 (1 %)

*MCTD* Mixed connective tissue disease, *SLE* systemic lupus erythematosus, *RA* rheumatoid arthritis, *SSc* systemic sclerosis, *SjS* Sjögren’s syndrome, *aa* amino acid, *SARD* systemic autoimmune rheumatic disease

Data are presented as number (%)

**Fig. 2:**
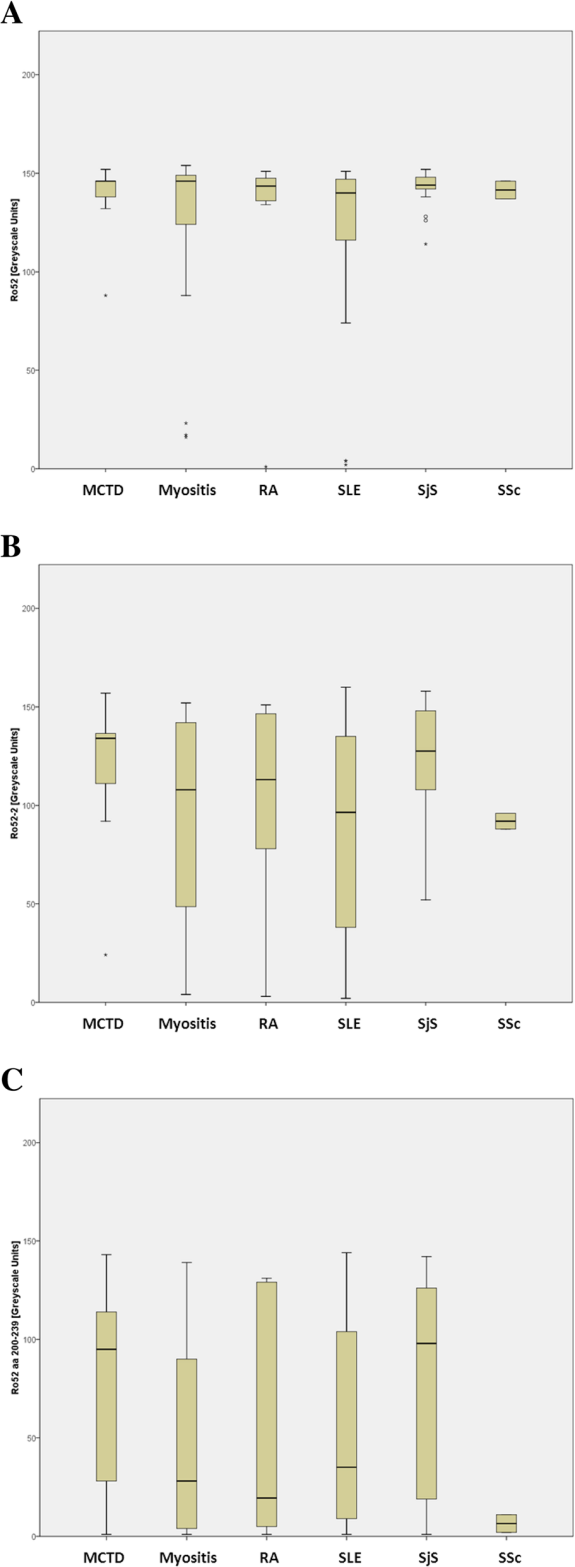
Boxplots (median [interquartile range]) representing distribution of antibody values (grayscale units) in the different systemic autoimmune rheumatic disease (SARD) cohorts for full-length Ro52 (**a**), fragment Ro52-2 (**b**), and Ro52 200–239-aa peptide (**c**). *aa* amino acid, *MCTD* mixed connective tissue disease, *RA* rheumatoid arthritis, *SjS* Sjögren’s syndrome, *SLE* systemic lupus erythematosus, *SSc* systemic sclerosis. Rings and stars indicate outliers and extreme values respectively

Quantitative antibody values did not differ among the SARD cohorts for fragments 1, 3, 4, and 5 or for the 175–196-aa peptide, which provided negative results. In our analysis of the distribution of the intensity of the positive reactions, we found that antibody levels did not differ substantially for the full Ro52 molecule, but they did for fragment 2 (103 ± 38 grayscale units) and for the 200–239-aa peptide (53 ± 49), which displayed a higher antibody level in the MCTD and SjS cohorts (Fig. 2). However, no statistically significant combination of antibodies against fragments and/or peptides implicated a disease compared with Ro52-2 antibodies marking a disease on its own (*p* < 0.001).

## Discussion

The most important findings of this study are that the main epitope of the Ro52 antigen was located in fragment Ro52-2 and that all patients with SARDs had antibodies that targeted this epitope. As the majority of the samples (97 %) were positive for Ro52-2, and only some for Ro52-4 or Ro52-5, it seems that the target epitope is localized in the middle of fragment Ro52-2 or in the area between Ro52-4 and Ro52-5. A very small number of samples were positive for Ro52-1 and for the 175–196-aa peptide; no samples were positive for fragment Ro52-3.

Anti-Ro52-5 antibodies were the second most frequent antibodies marking the 28 % of SARDs patients, while antibodies against the 200–239-aa peptide present on Ro52-5 and Ro52-2 marked the 74 % of patients with SARDs, even if in this cohort anti-Ro52-5 antibodies were sometimes not reacting.

In the area of the 200–239-aa peptide, leucine zipper structures are present. Leucine zippers are functional domains involved in protein–protein interactions and in dimer formations important for DNA binding. The strong positivity against the 200–239-aa peptide and the negativity for 175–196-aa peptide observed in all SARD sera indicate that the anti-Ro52 200–239-aa/176–196-aa signal intensity ratio is not a marker of a specific SARD cohort. Thus, the initial observation that the ratio constitutes a relevant marker for predicting fetal CHB and/or cardiac conduction defects in anti-Ro52–positive pregnant women [17] cannot be generalized to a SARD population.

Because anti-Ro52 antibodies are not specific for any given autoimmune rheumatic disease, the main aim of this experimental study was to find a distinct epitope recognition profile for each SARD cohort. However, the mapping of the specificity of anti-Ro52 antibodies did not enable identification of a distinct profile in any SARD cohort, and any antibody combination had added value compared with anti-Ro52-2 positivity alone. Only in patients with SjS did we observe a copresence of anti-Ro52-2 positivity with anti-Ro52-5 (38 %) or anti-Ro52-4 (15 %) antibodies.

These observations indicate that anti-Ro52-2 was the main and most important epitope in each single-SARD cohort. However, we must acknowledge that an important limitation of this exploratory study resides in the small number of patients included, especially in some SARD cohorts, such as SSc and MCTD. Another limitation of the study is that our disease control group did not include anti-Ro52–positive samples from patients with autoimmune diseases other than SARDs (e.g., patients with autoimmune liver diseases). Indeed, studies involving a disease control group of anti-Ro52–positive patients could help to show differences in epitope prevalence between SARD and non-SARD groups. Furthermore, because we did not have information on clinical features for most of the study patients, we were not able to analyze the serological findings in relation to clinical data. On the basis of these preliminary data, and given the potential significance of the present study, future studies with larger cohorts should be done to confirm the prevalence rates we found and to evaluate Ro52 epitope association with clinical parameters.

## Conclusions

The main epitope of the Ro52 antigen is localized on fragment 2 (125–267 aa). As most of the samples were positive for fragment 2 and only some were positive for fragments 4 or 5, which partially overlap fragment 2, it seems that the target epitope is localized in the middle of fragment 2 or in the area between fragments 4 and 5. The anti-Ro52 200–239-aa/176–196-aa signal intensity ratio does not represent a helpful tool to mark a SARD cohort, and no antibody combination was linked to any of the single SARD diseases. Future research is necessary to better define the clinical and serological characteristics of anti-Ro52–positive patients with SARD.

## Abbreviations

aa: amino acid
ANA: antinuclear antibody
CC: coiled-coil region
CHB: cardiac heart block
IFN: interferon
IIM: idiopathic inflammatory myopathy
MCTD: mixed connective tissue disease
RA: rheumatoid arthritis
SARD: systemic autoimmune rheumatic disease
SjS: Sjögren’s syndrome
SLE: systemic lupus erythematosus
SSc: systemic sclerosis

## References

[1] Ben-Chetrit E, Chan EK, Sullivan KF, Tan EM (1988). A 52-kD protein is a novel component of the SS-A/Ro antigenic particle. J Exp Med.

[2] Chan EK, Hamel JC, Buyon JP, Tan EM (1991). Molecular definition and sequence motifs of the 52-kD component of human SS-A/Ro autoantigen. J Clin Invest.

[3] Itoh K, Itoh Y, Frank MB (1991). Protein heterogeneity in the human Ro/SSA ribonucleoproteins: the 52- and 60-kD Ro/SSA autoantigens are encoded by separate genes. J Clin Invest.

[4] Boire G, Gendron M, Monast N, Bastin B, Ménard HA (1995). Purification of antigenically intact Ro ribonucleoproteins; biochemical and immunological evidence that the 52-kD protein is not a Ro protein. Clin Exp Immunol.

[5] Slobbe RL, Pruijn GJ, Damen WG, van der Kemp JW, van Venrooij WJ (1991). Detection and occurrence of the 60- and 52-kD Ro (SS-A) antigens and of autoantibodies against these proteins. Clin Exp Immunol.

[6] Dugar M, Cox S, Limaye V, Gordon TP, Roberts-Thomson PJ (2010). Diagnostic utility of anti-Ro52 detection in systemic autoimmunity. Postgrad Med J.

[7] Brouwer R, Hengstman GJ, Vree Egberts W, Ehrfeld H, Bozic B, Ghirardello A (2001). Autoantibody profiles in the sera of European patients with myositis. Ann Rheum Dis.

[8] Ghirardello A, Bassi N, Palma L, Borella E, Domeneghetti M, Punzi L (2013). Autoantibodies in polymyositis and dermatomyositis. Curr Rheumatol Rep.

[9] Ghillani P, André C, Toly C, Rouquette AM, Bengoufa D, Nicaise P (2011). Clinical significance of anti-Ro52 (TRIM21) antibodies non-associated with anti-SSA 60 kDa antibodies: results of a multicentric study. Autoimmun Rev.

[10] Liaskos C, Bogdanos DP, Rigopoulou EI, Norman GL, Shums Z, Al-Chalabi T (2007). Antibody responses specific for soluble liver antigen co-occur with Ro-52 autoantibodies in patients with autoimmune hepatitis [poster 660]. J Hepatol.

[11] Hervier B, Rimbert M, Colonna F, Hamidou MA, Audrain M (2009). Clinical significance of anti-Ro/SSA-52 kDa antibodies: a retrospective monocentric study. Rheumatology.

[12] Popovic K, Wahren-Herlenius M, Nyberg F (2008). Clinical follow-up of 102 anti-Ro/SSA-positive patients with dermatological manifestations. Acta Derm Venereol.

[13] Eriksson C, Kokkonen H, Johansson M, Hallmans G, Wadell G, Rantapää-Dahlqvist S (2011). Autoantibodies predate the onset of systemic lupus erythematosus in northern Sweden. Arthritis Res Ther.

[14] Frank MB, McCubbin V, Trieu E, Wu Y, Isenberg DA, Targoff IN (1999). The association of anti-Ro52 autoantibodies with myositis and scleroderma autoantibodies. J Autoimmun.

[15] Ghirardello A, Borella E, Beggio M, Franceschini F, Fredi M, Doria A (2014). Myositis autoantibodies and clinical phenotypes. Auto Immun Highlights.

[16] Salomonsson S, Dörner T, Theander E, Bremme K, Larsson P, Wahren-Herlenius M (2002). A serologic marker for fetal risk of congenital heart block. Arthritis Rheum.

[17] Ambrosi A, Wahren-Herlenius M (2012). Congenital heart block: evidence for a pathogenic role of maternal autoantibodies. Arthritis Res Ther.

[18] Bergman G, Eliasson H, Bremme K, Wahren-Herlenius M, Sonesson SE (2009). Anti-Ro52/SSA antibody-exposed fetuses with prolonged atrioventricular time intervals show signs of decreased cardiac performance. Ultrasound Obstet Gynecol.

[19] Defendenti C, Atzeni F, Spina MF, Grosso S, Cereda A, Guercilena G (2011). Clinical and laboratory aspects of Ro/SSA-52 autoantibodies. Autoimmun Rev.

[20] Oke V, Wahren-Herlenius M (2012). The immunobiology of Ro52 (TRIM21) in autoimmunity: a critical review. J Autoimmun.

[21] Burbelo PD, Ching KH, Han BL, Bush ER, Reeves WH, Iadarola MJ (2010). Extraordinary antigenicity of the human Ro52 autoantigen. Am J Transl Res.

[22] Bozic B, Pruijn GJ, Rozman B, van Venrooij WJ (1993). Sera from patients with rheumatic diseases recognize different epitope regions on the 52-kD Ro/SS-A protein. Clin Exp Immunol.

[23] Schulte-Pelkum J, Fritzler M, Mahler M (2009). Latest update on the Ro/SS-A autoantibody system. Autoimmun Rev.

[24] Ghirardello A, Rampudda M, Ekholm L, Bassi N, Tarricone E, Zampieri S (2010). Diagnostic performance and validation of autoantibody testing in myositis by a commercial line blot assay. Rheumatology (Oxford).

[25] Infantino M, Bentow C, Seaman A, Benucci M, Atzeni F, Sarzi-Puttini P (2013). Highlights on novel technologies for the detection of antibodies to Ro60, Ro52, and SS-B. Clin Dev Immunol.

